# Should We Use Rifampicin in Periprosthetic Joint Infections Caused by Staphylococci When the Implant Has Been Exchanged? A Multicenter Observational Cohort Study

**DOI:** 10.1093/ofid/ofae075

**Published:** 2024-02-08

**Authors:** Tobias Siegfried Kramer, Alex Soriano, Sarah Tedeschi, Antonia F Chen, Pierre Tattevin, Eric Senneville, Joan Gomez-Junyent, Victoria Birlutiu, Sabine Petersdorf, Vicens Diaz de Brito, Ignacio Sancho Gonzalez, Katherine A Belden, Marjan Wouthuyzen-Bakker

**Affiliations:** Institute for Hygiene and Environmental Medicine, Charité Universitätsmedizin Berlin, Berlin, Germany; Clinic for Orthopedic Surgery and Traumatology, Evangelisches Waldkrankenhaus Berlin, Berlin, Deutschland; LADR der Laborverbund Dr. Kramer & Kollegen, Geesthacht, Germany; Department of Infectious Diseases, University of Barcelona, IDIBAPS, Hospital Clinic of Barcelona, Barcelona, Spain; Department of Medical and Surgical Sciences, University of Bologna, Bologna, Italy; Infectious Diseases Unit, IRCCS Azienda Ospedaliero-Universistaria di Bologna, Bologna, Italy; Brigham and Women’s Hospital, Harvard Medical School, Boston, Massachusetts, USA; Infectious Diseases and Intensive Care Unit, Pontchaillou University Hospital, Rennes, France; French National Referent Centre for Complex Bone and Joint Infections, CRIOAC Lille-Tourcoing, Lille, France; Department of Infectious Diseases, Hospital del Mar, Infectious Pathology and Antimicrobial Research Group (IPAR), Institut Hospital del Mar d’Investigacions Mèdiques (IMIM), Universitat Autònoma de Barcelona (UAB), CEXS-Universitat Pompeu Fabra, Barcelona, Spain; Clinical Hospital of Orthopedics, Traumatology, and Osteoarticular TB, Bucharest, Romania; Institute for Medical Laboratory Diagnostics, Helios University Clinic Wuppertal, Wuppertal, Germany; Department of Infectious Diseases, Parc Sanitari Sant Joan de Deu, Sant Boi (Barcelona), Spain; Servicio de Cirugía Ortopédica y Traumatología, Hospital Universitario de Navarra, Pamplona, Spain; Division of Infectious Diseases, Sidney Kimmel Medical college at Thomas Jefferson University Hospital, Philadelphia, Pennsylvania, USA; Department of Medical Microbiology and Infection Prevention, University of Groningen, University Medical Center Groningen, Groningen, The Netherlands

To  The editor—We would like to thank the author for his interest in our research and for his comments regarding our article titled “Should We Use Rifampicin in Periprosthetic Joint Infections Caused by Staphylococci When the Implant Has Been Exchanged? A Multicenter Observational Cohort Study.”

We fully agree and are aware that small-colony variants (SCVs) can play an important role in staphylococcal periprosthetic joint infections (PJIs). Future studies should record this variable and investigate its impact based on species, chosen antibiotics, and outcome.

Clinical microbiologists should reach consensus on how to report information on SCVs in a structured form to clinicians. However, it is important to note that the absence of SCVs in culture does not rule out intracellular staphylococci.

In this study, we focused on cases of PJI that were treated with a total exchange of joint prothesis (1-stage or 2-stage) independant of a previous failure. The benefit of rifampicin was observed in patients with chronic infections treated with a 2-stage exchange. Total exchange followed by antibiotic treatment is widely accepted and used as a definitive treatment strategy. *S. aureus* was more frequent in the group not receiving rifampicin, and an infection with *S. aureus* that led to the explantation was identified as a independant risk factor for failure. The same is true for the use of rifampicin.

Our findings suggest that future studies should evaluate the additional effect on intracellular antibiotics (such as rifampicin) for PJI with *S. aureus* when no foreign bodies are present.

